# Infrared spectroscopy coupled to cloud-based data management as a tool to diagnose malaria: a pilot study in a malaria-endemic country

**DOI:** 10.1186/s12936-019-2945-1

**Published:** 2019-10-16

**Authors:** Philip Heraud, Patutong Chatchawal, Molin Wongwattanakul, Patcharaporn Tippayawat, Christian Doerig, Patcharee Jearanaikoon, David Perez-Guaita, Bayden R. Wood

**Affiliations:** 1 Centre for Biospectroscopy, School of Chemistry, Faculty of Science, Monash University, Wellington Road, Clayton, VIC 3800 Australia; 2 Department of Microbiology and the Biomedicine Discovery Institute, Faculty of Medicine, Nursing and Health Sciences, Monash University, Wellington Road, Clayton, VIC 3800 Australia; 3 Centre for Research and Development of Medical Diagnostic Laboratories, Faculty of Associated Medical Sciences, Khon Kaen University, Khon Kaen, Thailand; 4 Graduate School, Khon Kaen University, Khon Kaen, Thailand

**Keywords:** Malaria diagnosis, Infrared spectroscopy, Cloud based diagnostics, Plasmodium

## Abstract

**Background:**

Widespread elimination of malaria requires an ultra-sensitive detection method that can detect low parasitaemia levels seen in asymptomatic carriers who act as reservoirs for further transmission of the disease, but is inexpensive and easy to deploy in the field in low income settings. It was hypothesized that a new method of malaria detection based on infrared spectroscopy, shown in the laboratory to have similar sensitivity to PCR based detection, could prove effective in detecting malaria in a field setting using cheap portable units with data management systems allowing them to be used by users inexpert in spectroscopy. This study was designed to determine whether the methodology developed in the laboratory could be translated to the field to diagnose the presence of *Plasmodium* in the blood of patients presenting at hospital with symptoms of malaria, as a precursor to trials testing the sensitivity of to detect asymptomatic carriers.

**Methods:**

The field study tested 318 patients presenting with suspected malaria at four regional clinics in Thailand. Two portable infrared spectrometers were employed, operated from a laptop computer or a mobile telephone with in-built software that guided the user through the simple measurement steps. Diagnostic modelling and validation testing using linear and machine learning approaches was performed against the gold standard qPCR. Sample spectra from 318 patients were used for building calibration models (112 positive and 110 negative samples according to PCR testing) and independent validation testing (39 positive and 57 negatives samples by PCR).

**Results:**

The machine learning classification (support vector machines; SVM) performed with 92% sensitivity (3 false negatives) and 97% specificity (2 false positives). The Area Under the Receiver Operation Curve (AUROC) for the SVM classification was 0.98. These results may be better than as stated as one of the spectroscopy false positives was infected by a *Plasmodium* species other than *Plasmodium falciparum* or *Plasmodium vivax*, not detected by the PCR primers employed.

**Conclusions:**

In conclusion, it was demonstrated that ATR-FTIR spectroscopy could be used as an efficient and reliable malaria diagnostic tool and has the potential to be developed for use at point of care under tropical field conditions with spectra able to be analysed via a Cloud-based system, and the diagnostic results returned to the user’s mobile telephone or computer. The combination of accessibility to mass screening, high sensitivity and selectivity, low logistics requirements and portability, makes this new approach a potentially outstanding tool in the context of malaria elimination programmes. The next step in the experimental programme now underway is to reduce the sample requirements to fingerprick volumes.

## Background

Over 3.4 billion people are at risk of malaria, which remains one of the most devastating vector-borne diseases on the planet. It is also one of the world’s leading causes of childhood death [1]. Early detection and treatment is critical to saving lives and protecting the community from transmission of *Plasmodium* ssp. The focus on malaria prevention has now shifted to elimination of the disease, which the World Health Organization has set as a goal to achieve by 2030 [2]. Malarial elimination requires mass screening with diagnostics sensitive enough to detect asymptomatic carriers that act as reservoirs of infection. However, there is a lack of suitable inexpensive, highly sensitive and highly specific rapid diagnostic tests. Malaria diagnosis is currently reliant on rapid diagnostic tests (RDTs) based on antigen capture and immunochromatography, that do not attain the sensitivity and specificity required for effective malaria elimination [3, 4]. More sensitive approaches, such as reverse transcriptase polymerase chain reaction (RT-PCR), are time-consuming, require a large degree of technical expertise, expensive instrumentation and consumables, and are therefore not practical for malaria testing [3]. Light microscopy diagnosis also has sub-optimal sensitivity, and requires highly trained technicians, which has hampered implementation in resource-poor regions. Declining malaria transmission is accompanied by an increasing proportion of asymptomatic infections with extremely low, sub-microscopic parasite densities [5, 6]. This presents a major challenge for elimination, since a large proportion of the parasite reservoir is not detectable with the currently available tools; this problem exists for both *Plasmodium vivax* and *Plasmodium falciparum* [7]. A new approach for malaria diagnosis, that would be rapid, inexpensive, highly sensitive and selective, without the need for heavy logistics, would be a “game changer” in clinical diagnostics and public health surveillance, and would undoubtedly become a cornerstone of malaria elimination campaigns.

Fourier transform infrared (FTIR) spectroscopy is an emerging tool gaining ground in clinical diagnostics [8, 9]. It relies on the direct acquisition of the FTIR spectra from a biological sample without the need for any dye or reagent targeting the analyte or pathogen of interest [10]. When the energy of IR light matches the energy of vibrational transitions from a molecule or functional group an FTIR spectrum is generated, which is representative of the molecular composition of the sample [11, 12]. Multivariate data analysis methods can be employed to reveal changes in the spectra produced by variations in the biochemical composition that can be associated with changes of the phenotype caused by the presence of a pathogen [13, 14]. In 2014, a foundation paper for the detection and quantification of malaria parasite-infected cells using Attenuated Total Reflection-Fourier transform infrared (ATR-FTIR) spectroscopy on spiked red blood cell samples was published [15]. The technique and algorithms enabled the detection of early stage ring and gametocyte forms of the parasite to a level of detection 100 times lower than that of the current gold standard microscopy-based testing (i.e., able to detect a parasitaemia of 0.00001%). The method was also able to quantify parasitaemia levels with a quantification sensitivity limit of 0.1% with a 0.05 standard deviation. The approach combines the ability to detect malaria life stages that are found in the peripheral blood with rapid time-to-result, minimal sample preparation and portability of the device.

The next phase is to determine whether the approach can be adapted to a field setting, by comparing results of patient-derived clinical samples with parasite-spiked red blood cells under laboratory conditions as previously reported [15]. To this end, an assessment of the technology under clinical field conditions in Thailand, which included symptomatic patients with suspected malarial infection, was undertaken. The approach was optimised for non-expert users in remote settings by using quality control software that detects potential problems, such as spectral contamination from water vapour, poor absorbance and solvent contamination by cleaning agents. This ensured that only good quality spectra were presented to the model and subsequently processed. To facilitate translation of the technology, a “Cloud”-based system, with diagnostic algorithms located in secure remote servers was developed. This (i) protects the valuable models and algorithms from theft and retro-engineering of the device, and (ii) enables acquisition of databases that include de-identified patients, as well as GPS and spectral data with an epidemiological value. Users receive a simple diagnostic result without the need for any knowledge of spectroscopy or interpretation of the spectral data.

## Methods

### Sample collection, preparation and processing

Herein, we report the information regarding the study following the STARD (Standards for Reporting Diagnostic accuracy studies, see Additional file 1) [16]. Blood samples were acquired from regional clinics located in Thailand: Sunpasittiprasong Hospital, Ubonratchathani (UB); Srinagarind Hospital, Khon Kaen (MN); Kap Choeng Hospital, Surin (KS); Phop Phra Hospital, Tak (PP). This was covered by Khon Kaen University ethics applications HE 591238 and 033/2559. All patients presented with symptoms characteristic of suspected malaria infection. All patients were diagnosed for malaria at point of care by the hospital staff present using a combination of RDT and microscopy or microscopy alone. 3 mL EDTA blood was collected from each patient, to provide sub-samples for subsequent analysis at Khon Kaen University. The sample was used to prepare thick and thin blood smears for malaria examination by microscopy. 500 µL of packed red cells were aliquoted and resuspended into 4.5 mL absolute methanol (Additional file 2: Fig. S1). All samples were kept at 4 °C and transported to Khon Kaen University within 24 h (Additional file 2: Fig. S1 summarizing sample preparation steps). Samples were kept at 4 °C until analysis. Preliminary tests showed that samples could be stored at 4 °C for months without any appreciable change in the resulting ATR-FTIR spectra. Further details about the demography of the participants can be found in Additional file 3.

### Conventional malaria diagnostics at collection site

Blood smears from all patients were stained with Wright-Giemsa and used to diagnose malaria species by morphology under a light microscope. EDTA blood was checked for *P. falciparum (Pf)* and Pan (non-*Pf*) infections using SD Bioline malaria antigen strip (SD Diagnostics Inc., Republic of Korea) as rapid diagnostic test (RDT) according to manufacturer protocol.

### Reanalysis of blood smears by light microscopy

Blood smears of all collected samples (both positive and negative) were independently checked for the presence of *Plasmodium* species by 3 trained medical technologists at Srinagarind hospital, Khon Kaen University. A magnification of 1000× was used with 50 microscopic field typically examined.

### Identification of *Plasmodium* spp. using real time-PCR

#### Preparation of the DNA template

DNA was extracted from packed red cell preserved in methanol by QIAamp^®^ DNA Blood Mini Kit (QIAGEN, Germany) following the manufacturer’s ‘spin protocol’ procedure. In brief, 200 µL of lysis buffer containing 20 µL of QIAGEN protease solution was added into 200 µL of packed red cells. The mixture was incubated at 56 °C for 10 min and subsequently put through a spin column. Absolute ethanol was then used to wash and elute DNA. Eluded DNA was estimated for DNA concentration by spectroscopy measurement (Nano drop 2000, Wilmington, USA) at OD 260 nm. DNA samples were kept at − 20 °C until used.

#### Analytical performance of qPCR

Plasmids containing PCR products of 18s rRNA gene *P. falciparum* and *P. vivax* were used as standards. Plasmid DNA was diluted with sterile water to obtain 10^3^ to 10^8^ copies and used to establish a calibration curve for measuring *P. falciparum* and *P. vivax* by real time PCR using a TaqMan^®^ probe assay. The oligonucleotide sequences of primers and probes followed Perandin et al. [17], and purchased from BIONEER Corp., Korea (Additional file 4: Table S1). Fifty microliters of PCR mixture containing 1× of AccuPower^®^ Plus DualStar qPCR MasterMix (BIONEER Corp.), 0.3 µM of primers and probes and 1× of Rox dye. Amplification was initiated at 95 °C for 5 min, followed by 45 cycles at 95 °C for 15 s and 60 °C for 60 s using Exicycler™ 96 (BIONEER Corp.). The detection range of *P. falciparum* and *P. vivax* was estimated from standard curves.

Low (10^3^ copies), medium (10^4^ copies) and high (10^8^ copies) concentrations of both *P. falciparum* and *P. vivax* were used as internal controls for each run. Analytical precision was estimated from 3 levels of controls. The coefficient of variation (CV) represented the qPCR precision that were tested by using *P. falciparum* and *P. vivax* plasmids as the internal control materials for each run indicating repeatability of results in different runs.

CV was obtained from following formula

$${\text{CV}}\, = \,\left[ {{\text{Standard Deviation}}/{\text{Mean}}} \right]\, \times \, 100.$$

Analytical imprecision less than 15% CV was accepted for within runs and between runs. Reaction lacking DNA template was used as negative control in each batch. Cut-off values were selected according to the recommendations of the manufacturer.

### Malaria diagnosis using ATR-FTIR spectroscopy

#### ATR-FTIR spectroscopy data acquisition

The methodology for data acquisition using ATR-FTIR spectroscopy from methanol fixed packed red blood cells was identical to that previously described [15], except that a portable Agilent 4500 ATR-FTIR spectrometer was employed. IR spectra were acquired over the range from 4000 to 650 cm^−1^ at a spectral resolution of 4 cm^−1^, using 64 co-added interferograms for the background measurement and 128 co-added interferograms for the sample measurements. Background measurements were acquired in air before every new sample measurement following cleaning of the ATR crystal with absolute ethanol.

#### In-house quality control software

A methodology was established to perform quality control of the spectrum prior to the inclusion in the calibration set or the malaria prediction model. This ensures that an acquired spectrum has features similar to the features included in the model. It also ensures that technical issues are not going to interfere in the extraction of information from the model. For example, the following two methods of quality control were developed.

#### Quality controls (QCs) independent of the model (only dependent of the database)

The QCs endeavour to monitor excess (or deficit) of the different components and interferences included in the sample. The component relative concentration was estimated using the height of a band (with two minima baseline points) of the spectra specifically assigned to the specific component. This relative concentration, i.e. the height of a band, was compared to a distribution of relative concentration values of the components in the database. If the relative concentration fell inside the distribution, the spectrum was considered to be acceptable, otherwise the spectrum was rejected and measured again until it met all the criteria. To this end, a threshold was defined from the distribution tails as depicted in Additional file 5: Fig. S2, using the average and the standard deviation of the distribution values.

Three components were studied sequentially. Additional file 4: Table S2 summarizes the pre-processing used prior to the calculation of the relative component concentration, the band used for calculating the concentration, and the thresholds established. The three components were:

(i) Water vapour interferences (excess and deficit): Fluctuation in IR active atmospheric water between the background and sample measurements can cause negative and positive bands, which are detected by using positive and negative thresholds.
(ii) Methanol (excess): Methanol was used for fixing the samples and sometimes the drying process was not completely effective and some methanol remained.
(iii) Sample absorbance (deficit): Due to an irregular distribution of the RBCs between the clamp and the ATR crystal, in some cases there was not enough sample in contact with the crystal and the absorbance was too low.

#### Quality controls associated to the model

After a spectrum passed the quality controls (QCs) for water vapour, methanol and sample absorbance, it was projected onto the selected PLS-DA model. For the validation process, any result was rejected if it showed both, Hoteling’s T^2^ and SQ residuals values outside of the 95% of confidence interval. For the calibration process, if the spectrum showed significantly high Hoteling’s T^2^ and SQ residuals, it was investigated for any spectral anomaly and if none was found, it was included in the database for extending the representativeness of the calibration set.

#### “Cloud”-based data processing

The cloud system was used for two main proposes: (1) The creation of the dataset; and (2) the analysis and diagnostic output of samples.

### Creation of the dataset

First, the aim was to construct a database from spectra of high quality. This goal was challenging because the acquisition of the spectrum is designed to be performed in remote locations by staff untrained in spectroscopy and multivariate analysis. A *MATLAB*-based graphical user interface (GUI) was designed for the collection of the dataset. The user introduces the spectrum file into the GUI, which performed the different QCs of the spectra outlined in “Background” section. Prior to the QCs, the GUI loads the database from the Cloud to have access to the thresholds and the current PLS-DA model for the last QC (see Additional file 6: Fig. S3). If the spectra passed all the QCs it was included in the Cloud database. The PLS-DA model used on the “distance to the model” QC was a non-optimized preliminary PLS-DA model performed with the whole database, using first derivative (9 smoothing points), SNV, Pareto scaling and mean centring as the pre-processing steps and the 1800–800 cm^−1^ and the 3200–2800 cm^−1^ regions. For each new set of samples the PLS-DA model was updated. For the first 100 samples, the QCs were deactivated because there was not a database to compare. Along with the spectra, the GUI enabled one to load other sample parameters (Microscopy result, RDT result, PCR result, age, gender and other clinical parameters) to the dataset.

### Calibration, optimization and test of SVM and PLSDAs model

Additional file 7: Fig. S4 depicts the process employed in the creation, selection and testing of the PLS-DA and SVMs models. The whole routine aimed to select the best conditions in terms of pre-processing and spectral regions before the independent assessment of the classification performance with an external dataset:

(i) Samples were split between a calibration and validation dataset. To ensure representative calibration and validation subsets, the dataset was spit according to the Kennard-Stone [18] algorithm. In each classification 70% of the samples were used for calibration and the other 30% composed the external dataset. This set was ignored in the subsequent selection of the model.
(ii) Calibration samples were used for creating multiple PLS-DA and SVM models combining different pre-processing and spectral regions. The spectral pre-processing steps employed were: (1) first derivative, standard normal variate and mean centred; (2) second derivative, standard normal variate and mean centred; (3) first derivative, standard normal variate, Pareto scaling and mean centred; and (4) second derivative, standard normal variate, Pareto scaling and mean centred. The Savitzky Golay algorithm was employed for the derivation (Polynomial degree = 2), but for each pre-processing 7, 11 or 15 smoothing points were used in the derivative, giving 12 different pre-processing step combinations trialed. In addition, the following spectral regions were employed: 3140–3000, 2999–2770, 1784–1589, 1588–1470, 1469–1355, 1354–1186, 1185–1006, 1005–700 cm^–1^, giving 3060 combinations of different regions used in the models. In total 3060 (255 combination of spectral regions combined with 12 different pre-processing combinations) SVMs and 3060 PLSDA models were performed, monitoring the CV error for each model. In the case of the PLS-DA, the minimum cross validation error using a maximum of 10 latent variables was monitored for each model.
(iii) Models were sorted according to their cross-validation error and from the 10 models with the lowest cross validation error selected as the optimum model. This final selection was performed according to both complexity (a reasonable number of latent variables (LVs) in the case of tie PLS-DA) and interpretability of the regions selected. Positivity cut-offs were selected considering the lowest number of misclassified samples in the CV.
(iv) The classification performance of the final model was tested using the independent test set.

## Results

### Development of “Cloud”-based diagnostic system for non-expert users

Two portable Agilent Technologies 4500 ATR-FTIR spectrometers (Santa Clara, USA) were employed. These devices can be operated from a laptop computer or a mobile telephone with in-built software that guides the user through the simple measurement steps. For the creation of the database, ATR-FTIR spectra were uploaded to the Internet and processed on a remote server located at Monash University (Melbourne, Australia). Information pertaining to the patients including location based on the Global Positioning System (GPS) were uploaded simultaneously with the spectral data from the sample. The latter were passed through a series of quality tests that rejected spectra based on (i) possible contamination by sample fixation, (ii) water vapour absorbance, or (iii) spectral dissimilarity from the database (Additional file 5: Fig. S2). Data rejection was relayed back to the user interface, identifying the cause of the problem and requesting another spectrum to be acquired. 2% of measured samples were rejected. The database was used to create multivariate data analytic models based on partial least squares-discriminant analysis (PLS-DA) and support vector machine (SVM) classifiers, which predict if a sample is malaria positive, or negative according to the spectrum. The database was split into calibration and test datasets. Calibration samples (70%) were used to develop several models using different pre-processing and spectral regions. Venetian blind (10 splits) cross-validation was used to select the best models, which were then tested using the independent test set (30%). Data treatment was performed in Matlab from MathWorks (Natick, USA) using in-house written routines based on functions from the PLS toolbox from eigenvector Research (Manson, USA). Routines are available on request.

**Fig. 1:**
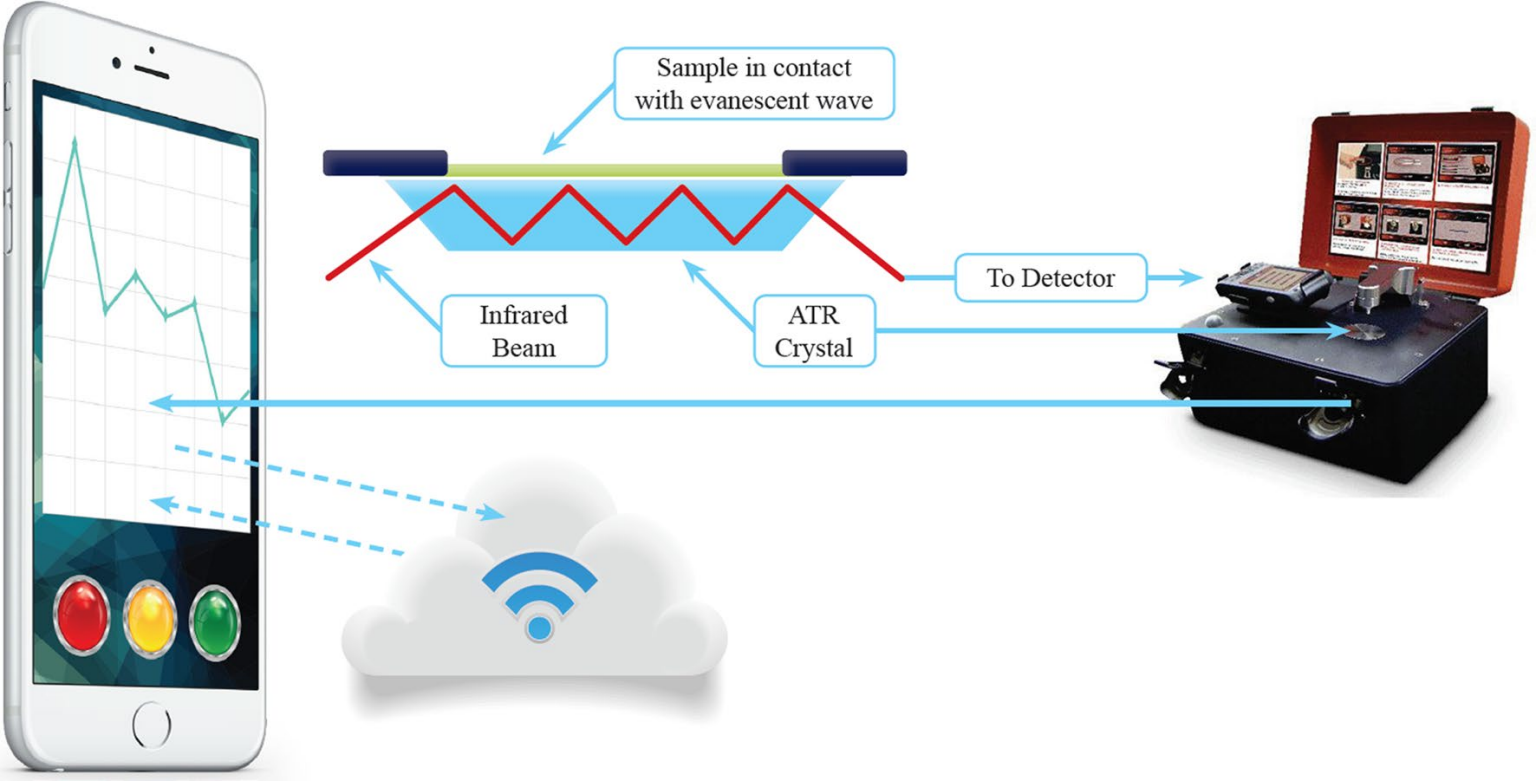
Portable “Cloud”-based diagnostic for malaria consisting of a portable ATR-FTIR spectrometer with an attached mobile telephone. The blood sample is placed onto the top of the ATR crystal and the IR evanescent wave penetrates a few microns and interacts with the sample, resulting in an infrared absorbance spectrum. This is conveyed (along with other data such as patient information and GPS position) to a secure remote server, where the spectrum is quality-checked and processed. A diagnostic algorithm finally determines a diagnostic result, which is sent back to a mobile telephone. See text for details

Once the classifiers are created, an unknown sample can be measured on site. The acquired spectra can be then encrypted and processed using the classifier located in the “Cloud”. After passing the quality test, the data are then: (i) pre-processed to minimize spectral baseline distortions, (ii) normalized to account for differences in pressure on the sample, and (iii) classified as either malaria-positive or -negative by the classification model. The result is then transferred back to the user’s mobile interface. The whole process from depositing the blood sample onto the spectrometer measurement window to receiving the diagnosis takes less than 5 min (see Fig. 1). Although, in the case of the study the cloud system was used by the researchers of the central laboratory in Australia, it can be applied on-site given an available internet connection.

### Summary of diagnostic results using RDT and light microscopy against gold standard qPCR

qPCR was able to detect between 10 to 10^7^ parasites per µL of blood, with average imprecision for *P. falciparum* and *P. vivax* at 9.85 and 10.08% coefficient of variation [CV; = (standard deviation/mean) × 100)], respectively. The between run precision calculated from the average CV across low (10^3^ copies), medium (10^4^ copies) and high (10^8^ copies) concentration for *P. falciparum* and *P. vivax* were 13.33 and 12.90%, respectively (Additional file 8: Fig. S5).

qPCR was used as a gold standard against which the performance of all diagnostic tests (rapid diagnostic test (RDT); light microscope; and, infrared spectroscopy) were compared. The comparison results of diagnostic using RDT and light microscopy against qPCR are summarized in Additional file 4: Table S3.

For the 318 samples tested, 197 were also analysed by standard serological RDT at point of care. Compared to PCR, there were 2 false positives and 1 false negative (1% and 0.5%, respectively). The two false positive samples displayed and RDT result as PAN (another species other than *P. falciparum* or *P. vivax*), whereas PCR was negative for both *P. falciparum* and *P. vivax.* It is possible that another *Plasmodium* species such *Plasmodium ovale* or *Plasmodium malariae*, that are found in the region, may have been present in these samples. The false RDT-negative sample was identified as *P. vivax* by PCR. For the 129 samples testing positive by both RDT and PCR, 87 identified the same species as PCR (14 *P. falciparum*; 67 *P. vivax*; 6 *P. falciparum*/*P. vivax*). Forty-two (32.6%) RDT-positive samples were misclassified with respect to species identification, compared to PCR: 36 samples were identified as PAN/*P. falciparum* whereas PCR identified all these as *P. falciparum*; 6 samples were identified as PAN by RDT and PCR identified 5 of these as *P. falciparum*/*P. vivax* and one as *P. falciparum.* The sensitivity and specificity of RDT test were 99.2% and 97%, respectively, when using PCR as a gold standard.

Light microscopy was used to examine thin films, using a “two out of three” consensus result from three trained microscopists. Compared with PCR, microscopy misidentified 5 samples out of 319; they were all false positive samples; 3 of 5 were identified as *P. falciparum* and other 2 samples for *P. vivax*, while PCR gave a negative result. For 162 negative and 131 positive samples (as tested by both light microscopy and PCR), 131 samples identified the same species as PCR (51 *P. falciparum*; 77 *P. vivax*; 3 *P. falciparum*/*P. vivax*). 20 samples identified as positive by light microscopy were misclassified with respect to species, as compared to PCR. 13 samples were identified as single infections, whereas PCR identified a mixed infection (both *P. falciparum* and *P. vivax*). 4 samples were identified as mixed infection, while PCR identified only *P. falciparum*, and 3 samples were misclassified (*P. vivax* by light microscopy, but *P. falciparum* by PCR). The sensitivity and specificity for light microscopy were 100% and 97%, respectively.

### Diagnostic results by infrared spectroscopy

**Fig. 2:**
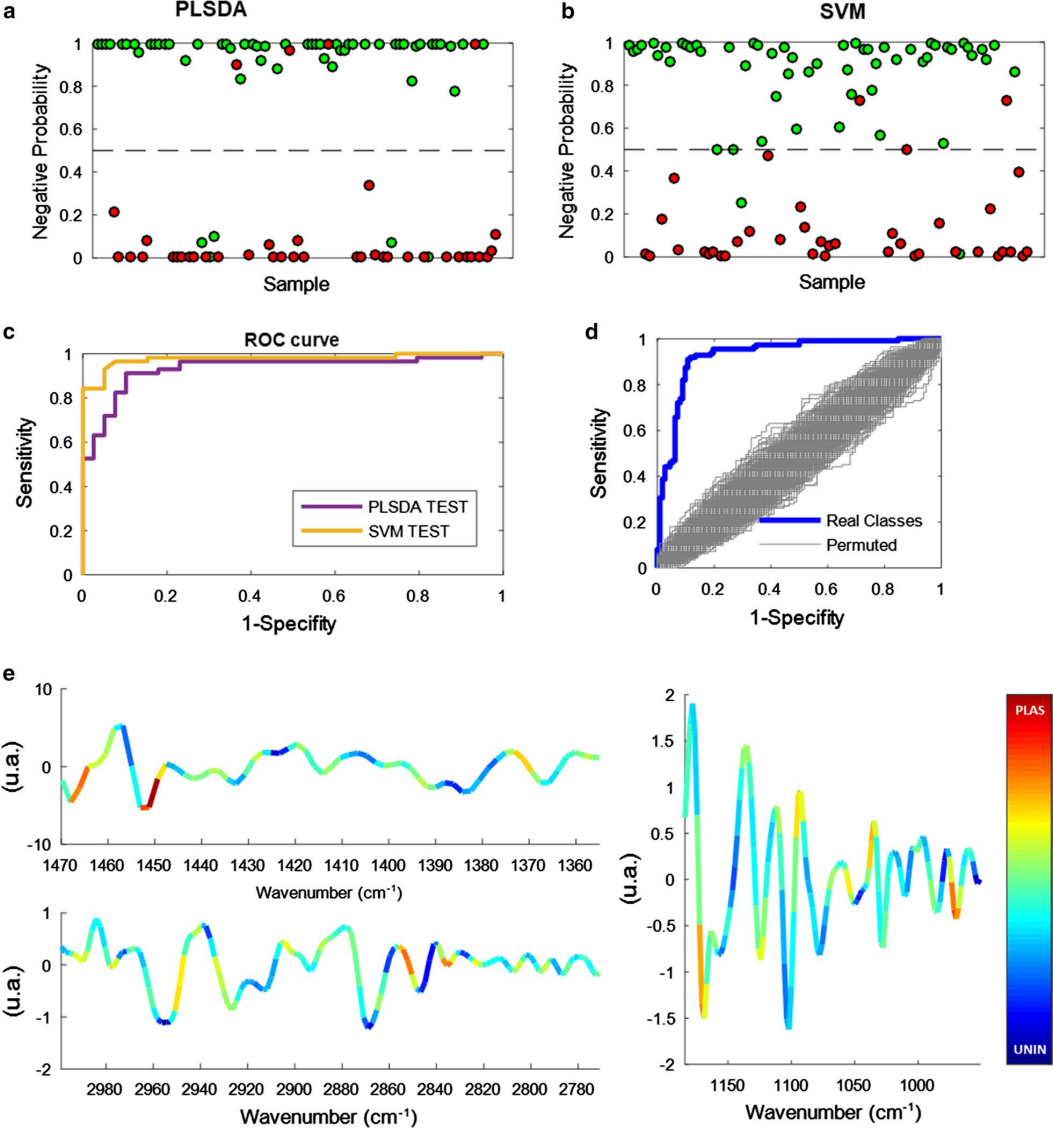
**a** Partial least squares discriminant analysis (PLS-DA) prediction plot showing the classification either malaria positive (< 0.5) or negative (> 0.5); spectra colour-coded malaria positive (red) or negative (green) by PCR. **b** Same as in a) except support vector machine (SVM) learning is used for the classification. **c** Receiver operating characteristic (ROC) curves showing the diagnostic of the PLS-DA and SVM classification. **d** ROC curve for data where samples were assigned positive- and negative, based on PCR versus randomized models. **e** Average spectra over the three spectral ranges used for PLS-DA classification. Superimposed is a colour code showing the regression loadings for malaria positive (“warm colours”) or negative (“cool colours”) classification for each absorbance value

All diagnostic modelling and validation testing was performed against the gold standard qPCR. Sample spectra from 318 patients were used for building calibration models (112 positive and 110 negative samples according to PCR testing) and independent validation testing (39 positive and 57 negatives samples by PCR). Various preprocessing methods and spectral regions were tested for the creation of the model and the parameters that minimized the cross-validation classification error were finally selected. Once the best model was selected, it was validated with an independent test set, which had not been used in the model development and optimization process. Panels 2a and 2b show the probability of the samples to be negative, being the samples coloured according to the PCA results. PLS-DA achieved 90% sensitivity (4 false negatives) and 91% specificity (5 false positives). SVM classification performed slightly better with 92% sensitivity (3 false negatives) and 97% specificity (2 false positives). The area under the receiver operation curve (AUROC) for the PLS-DA classification was 0.93 and for the SVM classification it was 0.98. A permutation test was performed to assess whether cross validation errors obtained in the modelling process were significant [19] (Fig. 2d). This test compares the classification accuracy figures obtained from the real classes compared to randomly permuted classes (null model). By doing so, one can assess if the errors obtained are different from classifications obtained by chance. In this study, the AUROCs obtained with the real classes were significantly higher than the ones obtained for the permuted classes (p < 0.05). The PLS-DA regression vector, which is indicative of the correlation (negative or positive) of the different IR variables to the presence of the infection, has been superimposed as a false-colour scale upon the mean raw spectrum from the study over the optimized spectral model ranges (Fig. 2e n = 319). These included infrared spectral ranges dominated by bands from: proteins and lipids (3140–2770 cm^−1^; 1470–1355 cm^−1^) and nucleic acids (1185–950 cm^−1^). The colour code is used to evidence the contribution of the different bands to the classification, being the “cold” or “warm” colors correlated to the absence or presence of the parasite, respectively. For example, the strong red coloration of the band at 1450 cm^−1^ indicates that this band is correlated to the presence of the parasite. This band is assigned to the deformation of the CH_2_ modes, present in lipids. Thus, this suggest that the model is using, among other molecular information, the presence of the lipids to identify the infection. Although the technique is label-free and the discrimination power is based on the changes on the metabolic profiles, the coloration of the bands evidences that the model uses changes on the general phenotype to predict the presence or absence of the parasite.

## Discussion

The paper by Khosmanesh et al. [15] demonstrated the potential of IR spectroscopy for ultra-sensitive detection of malaria parasites in spiked red blood cell samples, down to levels equivalent to PCR detection limits. The present study was aimed at establishing whether the methodologies developed under laboratory conditions can be translated to work with clinical samples in a remote regional setting. Part of the demands of this translation was the need to process spectral acquisition, quality control and diagnostics via “Cloud-based” data system.

The ATR-FTIR spectroscopy-based diagnostic demonstrated excellent sensitivity and specificity against the PCR gold standard. The support vector machine learning algorithm performed better than the PLS-DA modelling approaches (AUROC = 0.98 vs 0.93). Empirical testing of modelling performance based on sample numbers (Additional file 9: Fig. S6) indicated that sensitivity and specificity are likely to improve further with calibration set numbers up to n = 500. When applying the SVM classification there were three false negatives and two false positives. One case of misclassification could be accounted for; a false-positives classified positive by both microscopy and RDT (Pan). It appears this patient was infected by a *Plasmodium* species other than *P. falciparum* or *P. vivax*, not detected by RDT or by the PCR primers employed.

An examination of the regression vectors for the PLS-DA classification models generated in this study and those obtained from laboratory spiking experiments by Khosmanesh et al. showed that the same wavenumber regions were important for the positive classification of malaria in both the field and laboratory studies. In the laboratory study, the C-H stretching region (2990–2770 cm^−1^) was found to be crucial to discern infected from uninfected cells [15], which is explained by the fact that this region corresponds to neutral lipids specific to the malaria parasites. In the present study lower wavenumber spectral windows (1469–1355; 1185–1006 cm^−1^) were also included in the optimized PLS-DA regression vector, consistent with molecular changes known to occur as a consequence of infection. For example, the spectral region 1354–1186 cm^−1^ contains bands assigned to proteins. Protein levels in blood cells decrease in samples due to the consumption of haemoglobin by malaria parasites, and this is detected by spectroscopy [20]. The lower wavenumber region (1185–1006 cm^−1^) includes bands from nucleic acids shown in the laboratory studies [15, 20] to be strongly loaded in models used to discriminate malaria infected samples.

This study demonstrated the successful operation of a Cloud-based diagnostic system based on spectroscopy. The importance of this cannot be overestimated because it allows operation of the system by workers who are not trained spectroscopists. Including a quality control step in the workflow ensured that only good-quality spectra were added to the database to further refine the diagnostic model. Given that positional information is provided through GPS data, the system has potential for epidemiological analysis, and could be used to monitor disease distribution and outbreaks. It will also be possible to retrospectively mine the database for other diagnostic information that may exist in the spectral information [21]. A limitation of the system is that it relies on connectivity to a mobile telephone network or the Internet. This disadvantage is balanced by the greater commercial security for the diagnostic algorithms provided by an encrypted cloud-based system, and by the privacy of patient-related data it stores. The system can conceivably incorporate a “hold mode”, such that the system would save input data until connectivity to a telephone network or the Internet was established, and then upload the data for processing and diagnoses.

Other potential drawbacks have been identified. For example, it has been shown that anticoagulants used in blood collection tubes can affect ATR-FTIR spectroscopy-based malaria diagnosis [20], depending on the volume of blood that is drawn into the collection tube and the type of anticoagulant employed. Only one type of anticoagulant (EDTA) was employed in this study, which did not yield spectral bands that could confound malaria diagnosis. It is proposed that the volume of blood was sufficient to dilute the anticoagulants. The use of methanol to fix red blood cells followed the methodology established in the laboratory [15]. Fixing the samples provided a convenient method for ensuring consistent samples could be transported to the central processing station in Khon Kaen. However, this is not suitable for POC malaria diagnostics using ATR-FTIR spectroscopy. Ideally, a POC test should employ finger-prick blood volumes that can be measured directly at POC, rather than venous samples processed in a central laboratory as reported here. It has also been demonstrated [20] that malaria parasites can be detected with parasitaemia levels < 0.1% directly from unfixed aqueous blood samples. This approach, requiring less than 200 mL of blood, could easily be used in POC clinical settings, and would be particularly suited to diagnosing malaria in infants. Another relevant development is the ability to use spectroscopy to measure blood chemistry parameters such as haemoglobin and urea simultaneously with the malaria diagnostic measurement [21]. As patients infected with malaria often present with anaemia, an accurate, rapid and inexpensive POC determination of blood haemoglobin (Hb) levels would greatly facilitate patient care and management.

The laboratory study [15] in which the experimental procedure for this trial was developed achieved detection sensitivities similar to PCR-based approaches. This trial was not designed as a test of sensitivity per se, as this will be tested in future trials. An appropriate approach for specifically assessing the performance of the spectroscopic approach in detecting asymptomatic carriers would be to conduct tests in regions where malaria is endemic but the majority of people are asymptomatic. This possibility is being investigated in other malaria endemic regions, which fulfils the above criteria. The notion would be to test every member of a community (e.g. a village) with ATR-FTIR spectroscopy and PCR in parallel. Spectra of samples from asymptomatic (and symptomatic) carriers could then be used to develop calibration models and subsequent validation testing.

## Conclusions

Overall, it was demonstrated: (i) that ATR-FTIR spectroscopy has the potential to be developed as an efficient and reliable malaria diagnostic tool at POC under tropical field conditions; and (ii) that spectra could be analysed via a Cloud-based system, and the data fed back to the user; both aspects carry significant potential. The fact that the entire procedure requires only a portable spectrometer and a mobile phone makes mass screening a definite possibility. Indeed, the cloud-based analysis implies that the health care personnel do not need to be skilled in anything else other than collecting blood. Once the procedure has been optimized with blood drawn from a fingerprick rather than blood collected in tubes, the approach will be even easier to implement. The machine can be easily transported in a backpack, and power can be supplied from a portable/foldable solar panel, making the penetrance of the system into remote areas feasible. Connection to the cloud can be postponed after data acquisition. Centralized data analysis will allow investigators to shed light on the epidemiology of malaria and any other disease agents for which an IR signature is available.

The combination of accessibility to mass screening, high sensitivity and selectivity, low logistical requirements and portability, makes this approach a potentially outstanding tool in the context of malaria elimination programmes.

## Supplementary information

**Additional file 1: Text S1**. STARD Checklist.

**Additional file 2: Fig. S1.** Summary of the sampling methodology and experimental design.

**Additional file 3: Text S2**. Baseline demographic and clinical characteristics of participant.

**Additional file 4: Table S1**. Primers and probes of 18s rRNA malaria parasites according to Perandin et al. [17]. **Table S2**. Parameters of the QCs independents of the model used on this study. NOTE. SD: Standard deviation. **Table S3.** summarizing the comparison of malaria diagnosis by light microscopy and serological RDT against the gold standard real time-PCR analysis (a). Diagnostic sensitivity and specificity of RDT (b) and light microscopy (c) were calculated.

**Additional file 5: Fig. S2.** Scheme of the method for the quality controls of the spectrum. The relative concentration of the component in the spectrum is compared with the distribution of this value in the dataset. Thresholds are defined taking into account the average value and standard deviation of the distribution. If the relative concentration of the component in the spectra is outside of the threshold, the spectrum does not pas the QC.

**Additional file 6: Fig. S3.** Outlining software package developed in-house to process spectra received by the “Cloud” diagnostic system. Spectra are passed through a series of quality control steps, before being passed to the diagnostic model for classification.

**Additional file 7: Fig. S4.** Scheme representing the process of creation, optimization and testing of a model.

**Additional file 8: Fig. S5.** Analytical performance of qPCR for *P. falciparum* and *P. vivax.* (a) calibration data for qPCR with *P. falciparum;* (b) calibration data for qPCR with *P. vivax*; (c) Real time PCR precision data. Each measurement in the standard curves are the mean of 9 measurements resulting from three technical replicates within three independent experiments.

**Additional file 9: Fig. S6.** Using field data from the pilot trial the effect of sample size on classification accuracy was studied. Cross validation classification performance was monitored by cross validation using successive PLS-DA models with increasing number of samples in the calibration data sets (up to n = 200; using 20 replicates in each case). Classification error decreases exponentially with sample size (n). Extrapolation of the trend line predicts very low error rate at n = 500.

## Data Availability

The datasets used and/or analysed during the current study will be made available from the corresponding author on request.

## Abbreviations

POC: point-of-care
RDTs: rapid diagnostic tests
RT-PCR: reverse transcriptase polymerase chain reaction
FTIR: Fourier transform infrared
ATR-FTIR: Attenuated Total Reflection-Fourier transform infrared
STARD: Standards for Reporting Diagnostic accuracy studies
GUI: graphical user interface
PLS-DA: partial least square-discriminant analysis
QC: quality control
SVM: supported vector machines
LVs: latent variables
ROC: receiver operating characteristic
