# Supplementary figures and images for: Infrared spectroscopy coupled to cloud-based data management as a tool to diagnose malaria: a pilot study in a malaria-endemic country
Source: Malar J. 2019 Oct 16;18:348. doi: 10.1186/s12936-019-2945-1 (PMC6794904; doi:10.1186/s12936-019-2945-1)

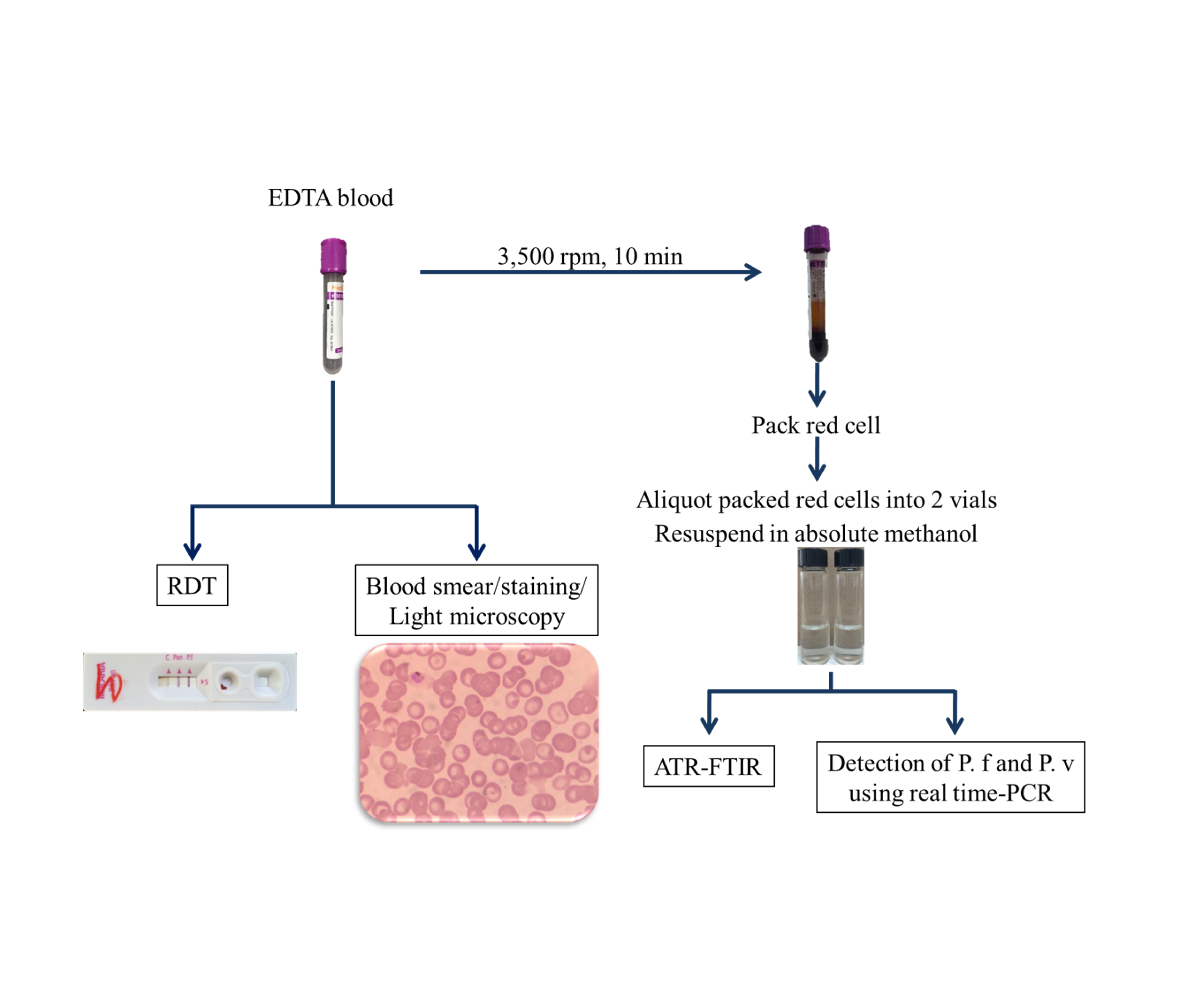

Supplement: Supplementary file 2 — Additional file 2: Fig. S1. Summary of the sampling methodology and experimental design. [file 12936_2019_2945_MOESM2_ESM.tif]

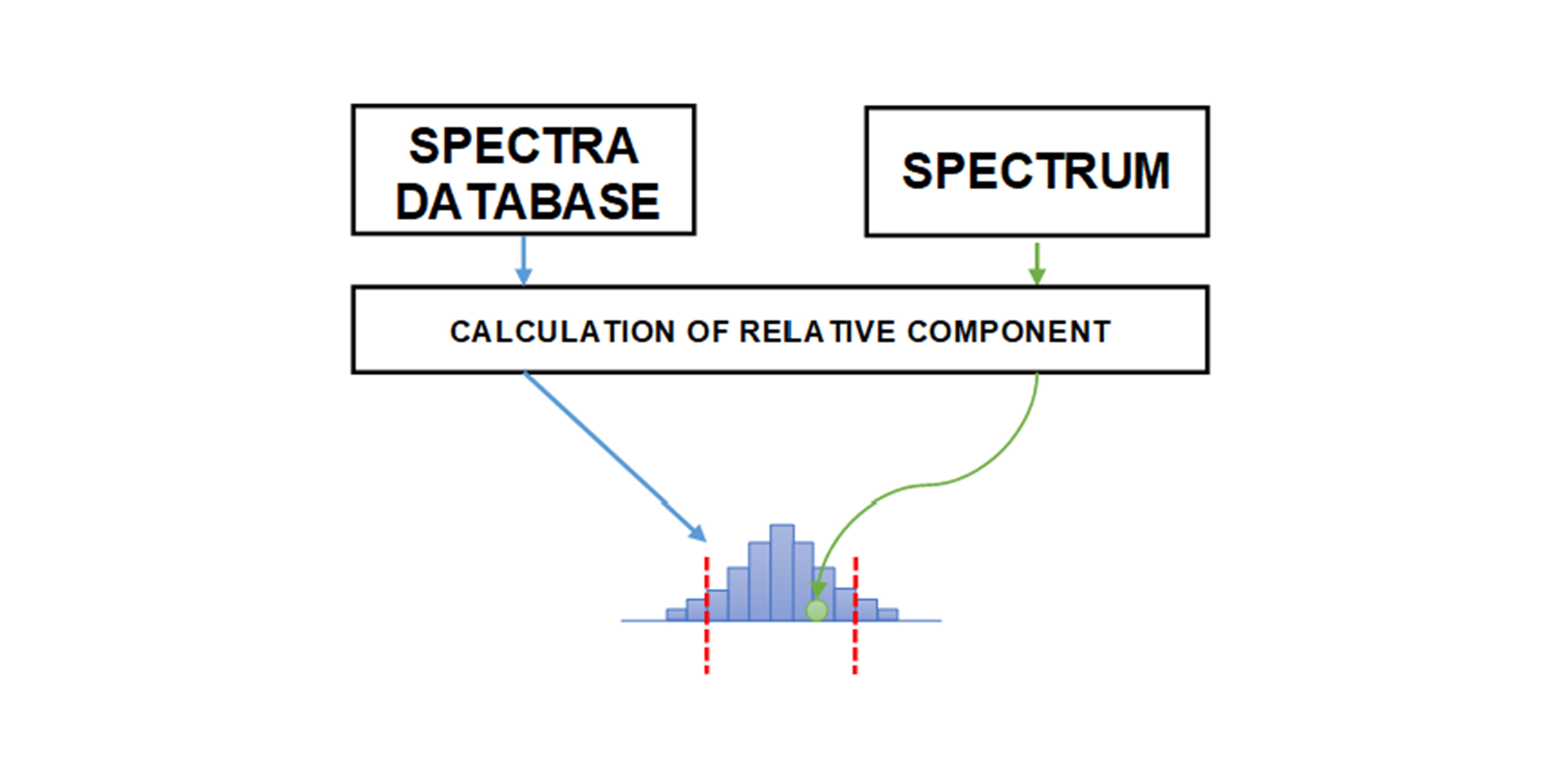

Supplement: Supplementary file 5 — Additional file 5: Fig. S2. Scheme of the method for the quality controls of the spectrum. The relative concentration of the component in the spectrum is compared with the distribution of this value in the dataset. Thresholds are defined taking into account the average value and standard deviation of the distribution. If the relative concentration of the component in the spectra is outside of the threshold, the spectrum does not pas the QC. [file 12936_2019_2945_MOESM5_ESM.tif]

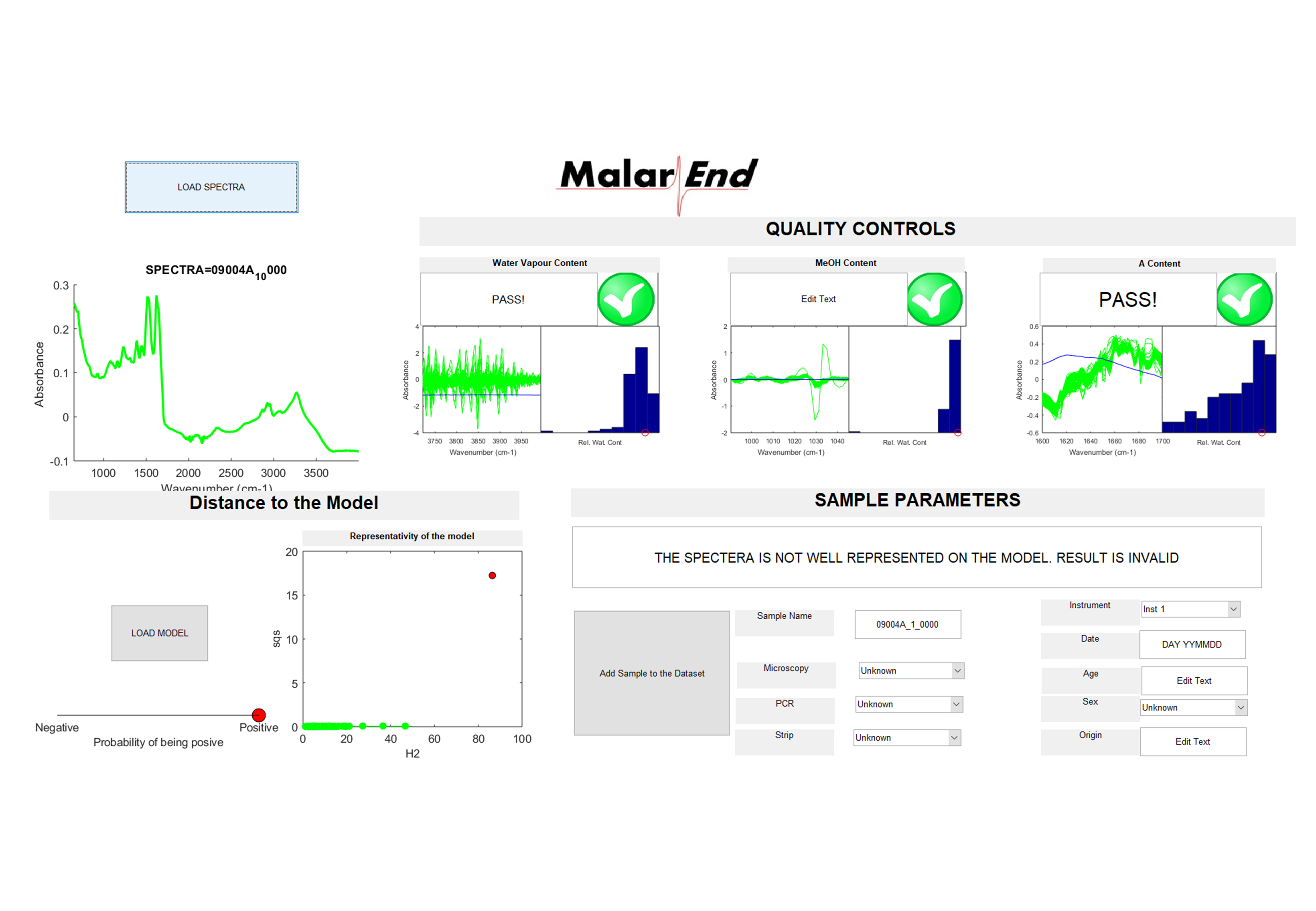

Supplement: Supplementary file 6 — Additional file 6: Fig. S3. Outlining software package developed in-house to process spectra received by the “Cloud” diagnostic system. Spectra are passed through a series of quality control steps, before being passed to the diagnostic model for classification. [file 12936_2019_2945_MOESM6_ESM.tif]

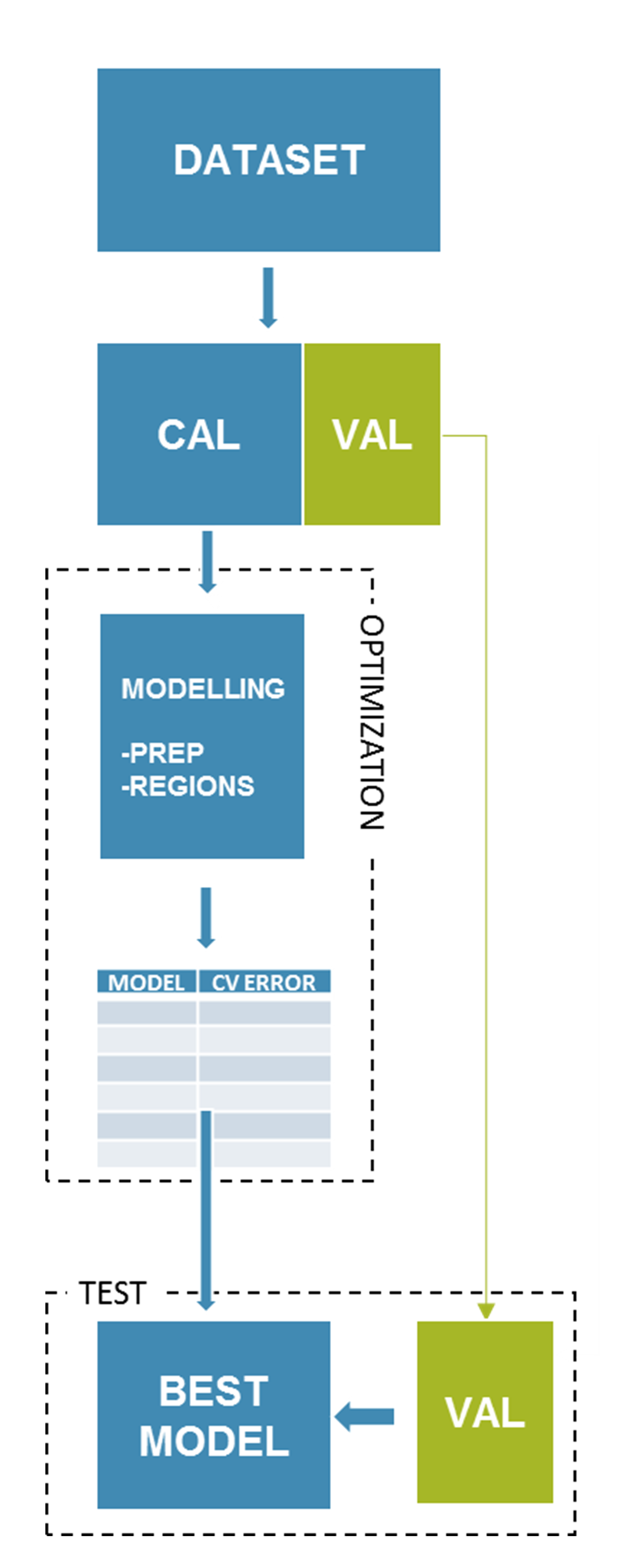

Supplement: Supplementary file 7 — Additional file 7: Fig. S4. Scheme representing the process of creation, optimization and testing of a model. [file 12936_2019_2945_MOESM7_ESM.tif]

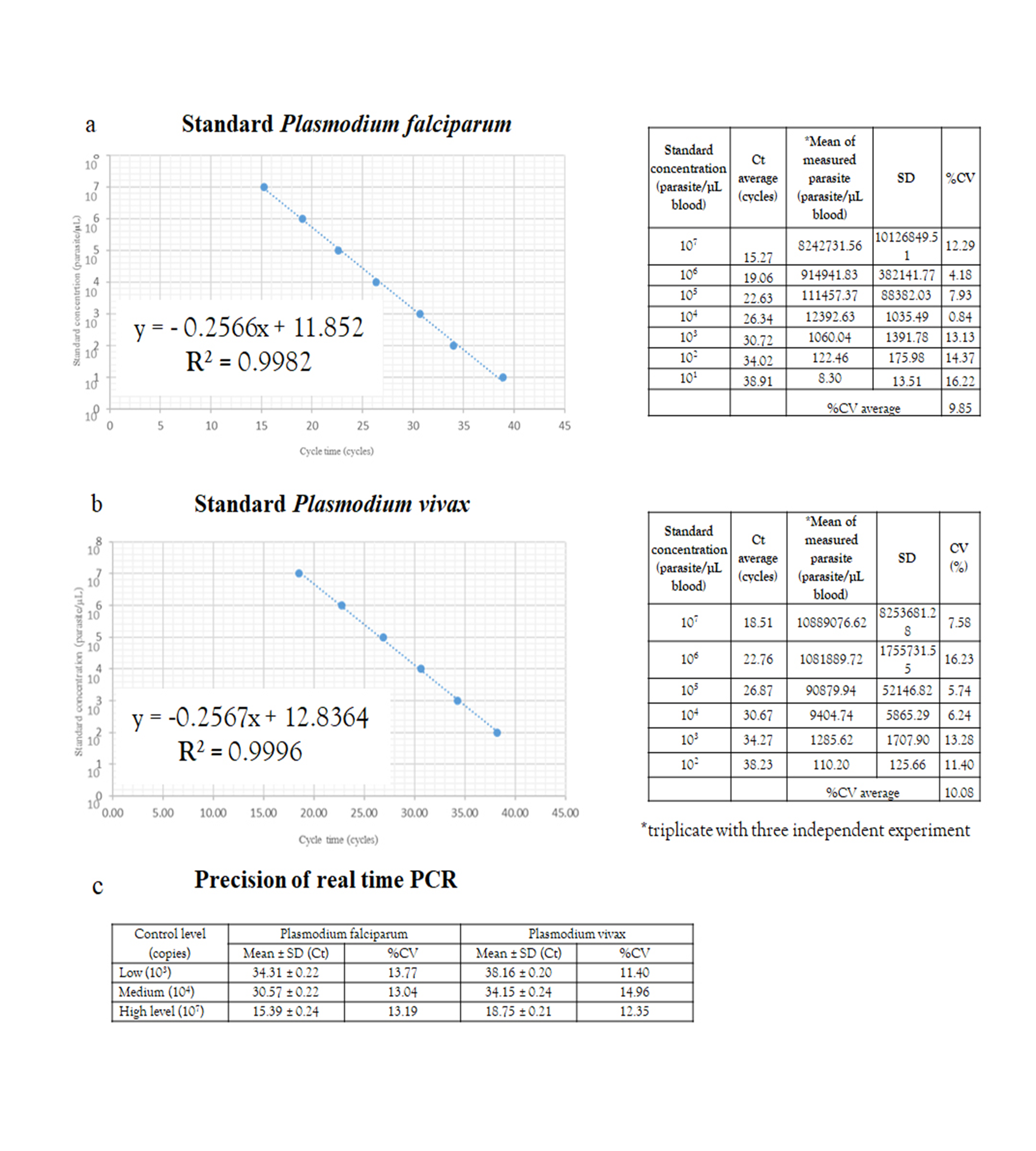

Supplement: Supplementary file 8 — Additional file 8: Fig. S5. Analytical performance of qPCR for P. falciparum and P. vivax. (a) calibration data for qPCR with P. falciparum; (b) calibration data for qPCR with P. vivax; (c) Real time PCR precision data. Each measurement in the standard curves are the mean of 9 measurements resulting from three technical replicates within three independent experiments. [file 12936_2019_2945_MOESM8_ESM.tif]

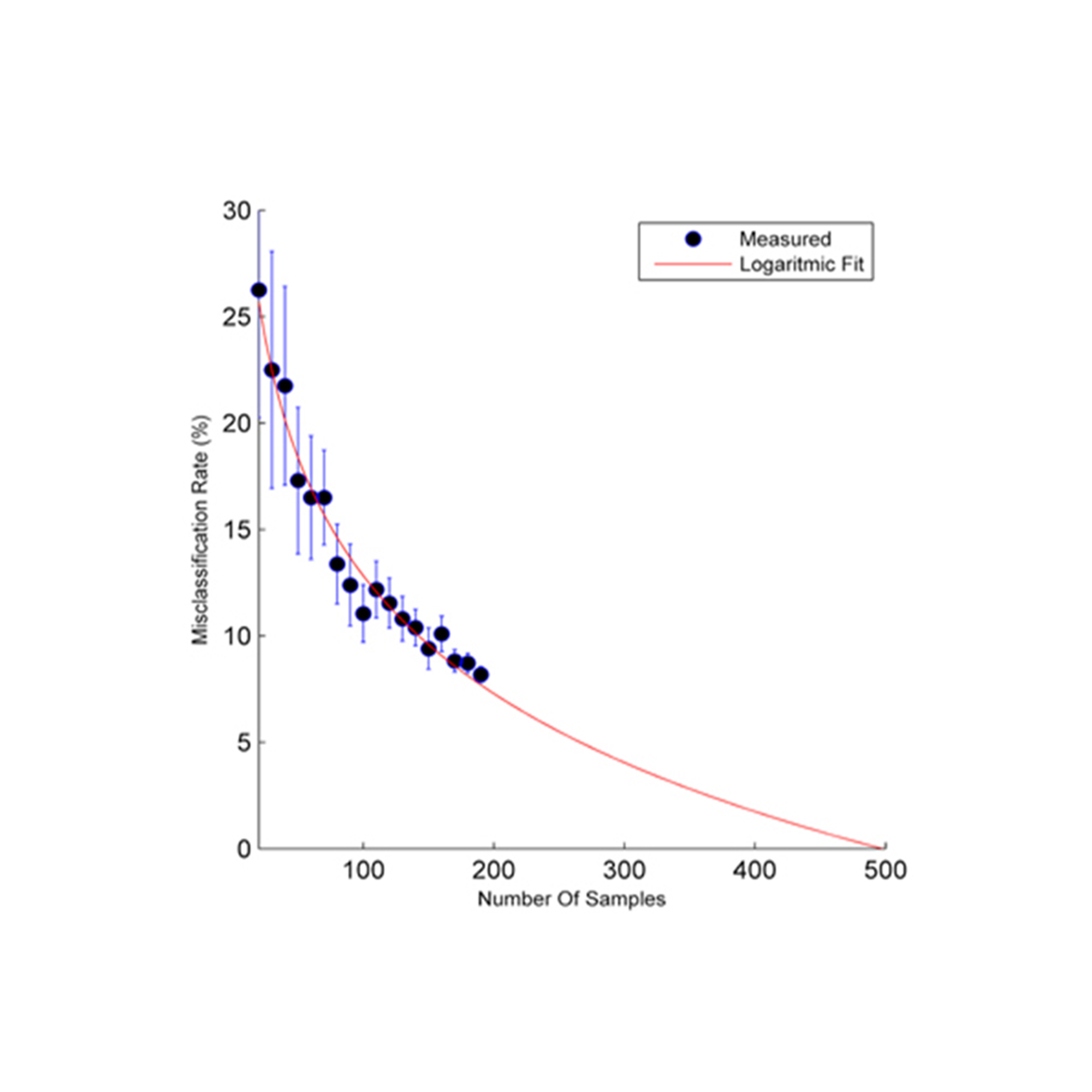

Supplement: Supplementary file 9 — Additional file 9: Fig. S6. Using field data from the pilot trial the effect of sample size on classification accuracy was studied. Cross validation classification performance was monitored by cross validation using successive PLS-DA models with increasing number of samples in the calibration data sets (up to n = 200; using 20 replicates in each case). Classification error decreases exponentially with sample size (n). Extrapolation of the trend line predicts very low error rate at n = 500. [file 12936_2019_2945_MOESM9_ESM.tif]
