# Supplementary material for: Infrared spectroscopy coupled to cloud-based data management as a tool to diagnose malaria: a pilot study in a malaria-endemic country
Source: Malar J. 2019 Oct 16;18:348. doi: 10.1186/s12936-019-2945-1 (PMC6794904; doi:10.1186/s12936-019-2945-1)
Supplement: Supplementary file 3 — Additional file 3: Text S2. Baseline demographic and clinical characteristics of participant. [file 12936_2019_2945_MOESM3_ESM.docx]

**Text S2: Baseline demographic and clinical characteristics of participant**

- **Table shows demographic data of participants**

| Details |  | PCR result | |
| --- | --- | --- | --- |
|  |  | Positive | Negative |
| Age (years) | Mean (min-max) | 35 (4-82) | 45 (1-84) |
| Sex (n) | Male : Female (ratio) | 120:31 (4:1) | 97:70 (1.4:1) |

- **Table shows origin data of participants**

| Collection site | Khon Kaen | 91 |
| --- | --- | --- |
|  | Ubon Ratchathani | 184 |
|  | Surin | 24 |
|  | Tak | 19 |
|  | Total | 151 |

- **Clinical characteristics were shown in Table S1**

|  | | | **PCR** | | | | **Total** |
| --- | --- | --- | --- | --- | --- | --- | --- |
|  |  |  | **Pos** | | | **Neg** |  |
|  |  |  | **P.f** | **P.v** | **Both P.f/P.v** |  |  |
| **Light microscopy** | **Pos** | **P.f** | 51 | 0 | 5 | 3 | 59 |
|  |  | **P.v** | 3 | 77 | 8 | 2 | 90 |
|  |  | **Both P.f/P.v** | 4 | 0 | 3 | 0 | 7 |
|  | **Neg** | | 0 | 0 | 0 | 162 | 162 |
| **Total** | | | 58 | 77 | 16 | 167 | 318 |
| **RDT** | **Pos** | **Pf** | 14 | 0 | 0 | 0 | 197 |
|  |  | **Pan** | 1 | 67 | 5 | 2 |  |
|  |  | **Pan/Pf** | 36 | 0 | 6 | 0 |  |
|  | **Neg** | | 0 | 1 | 0 | 65 |  |
|  | **Not analyzed** | | 7 | 9 | 5 | 100 | 121 |
| **Total** | | | 58 | 77 | 16 | 167 | 318 |

PCR: Polymerase Chain reaction, RDT: Rapid Diagnostic Test

**Distribution of severity of disease in those with the target condition**

**No data of severity was obtained.**

Patients suspected with malaria can present asymptomatically or symptomatically. Severe symptoms of malarial infection can include shock and coma in cerebral malaria, acute respiratory distress and pulmonary edema in the lung and impairment of renal function in the kidney; severe malaria symptoms are mostly associated with *Plasmodium falciparum* infection. Therefore, it is important to diagnose the species of *Plasmodium* infection, particularly as different *Plasmodium* species require different drug treatment. Distribution of *Plasmodium* spp. was shown in Supplementary Table 1.)

**Distribution of alternative diagnoses in those without the target condition**

At collection sites, RDTs were used as an alternative diagnosis for malarial infection in parallel with microscopic examination. All negative and positive cases (n=318) were confirmed by PCR at research site (Khon Kaen University).

- Malarial negative; 167 cases from collection site (Tak, Surin, Ubon Ratchathani and Khon Kaen)
- Malaria positive; 151 cases from collection sites

**Time interval and any clinical interventions between index test (IR) and reference standard (PCR)**

- Red blood cells and malarial contents were preserved in methanol at 4°C directly upon collection and transported at 4°C to research site within 24 hours.
- ATR-FTIR measurements and DNA extraction were performed on sub-samples from the methanol preserved material in parallel within 1 week of collection. Extracted DNA was stored at -20 °C until PCR measurements were performed.
