# Supplementary material for: Infrared spectroscopy coupled to cloud-based data management as a tool to diagnose malaria: a pilot study in a malaria-endemic country
Source: Malar J. 2019 Oct 16;18:348. doi: 10.1186/s12936-019-2945-1 (PMC6794904; doi:10.1186/s12936-019-2945-1)
Supplement: Supplementary file 4 — Additional file 4: Table S1. Primers and probes of 18s rRNA malaria parasites according to Perandin et al. [17]. Table S2. Parameters of the QCs independents of the model used on this study. NOTE. SD: Standard deviation. Table S3. summarizing the comparison of malaria diagnosis by light microscopy and serological RDT against the gold standard real time-PCR analysis (a). Diagnostic sensitivity and specificity of RDT (b) and light microscopy (c) were calculated. [file 12936_2019_2945_MOESM4_ESM.docx]

**Table S1**. Primers and probes of 18s rRNA malaria parasites according to Perandin et al.

| **Species** | **Primer and probe** | **Sequence** | **Product size** |
| --- | --- | --- | --- |
| *P. falciparum* | FAL-F  FAL-R  FAL probe | 5’-CTTTTGAGAGGTTTTGTTACTTTGAGTAA  5’-TATTCCATGCTGTAGTATTCAAACACAA  5’ FAM-TGTTCATAACAGACGGGTAGTCATGATTGAGTTCA-TEMRA | 98 |
| *P. vivax* | VIV-F  VIV-R  VIV probe | 5’-ACGCTTCTAGCTTAATCCACATAACT  5’-ATTTACTCAAAGTAACAAGGACTTCCAAGC  5’ TET-TTCGTATCGACTTTGTGCGCATTTTGC-TEMRA | 141 |

**Table S2**: Parameters of the QCs independents of the model used on this study. NOTE. SD: Standard deviation.

| *QC* | *Pre-processing* | *Calculation of relative concentration* | *Thresholds* |
| --- | --- | --- | --- |
| **H_2_O(g)** | Normalization | Abs at 3846 cm^-1^ – Abs at 3852 cm^-1^ | < Average - 1.5 SD  > Average + 1.5 SD |
| **MeOH** | First Derivative | Abs at 1029 cm^-1^ – Abs at 1033 cm^-1^ | > Average +1.5 SD |
| **Sample** | None | Absorbance at 1650 cm^-1^ | < Average -1.5 SD |

**Table S3** summarizing the comparison of malaria diagnosis by light microscopy and serological RDT against the gold standard real time-PCR analysis (a). Diagnostic sensitivity and specificity of RDT (b) and light microscopy (c) were calculated.


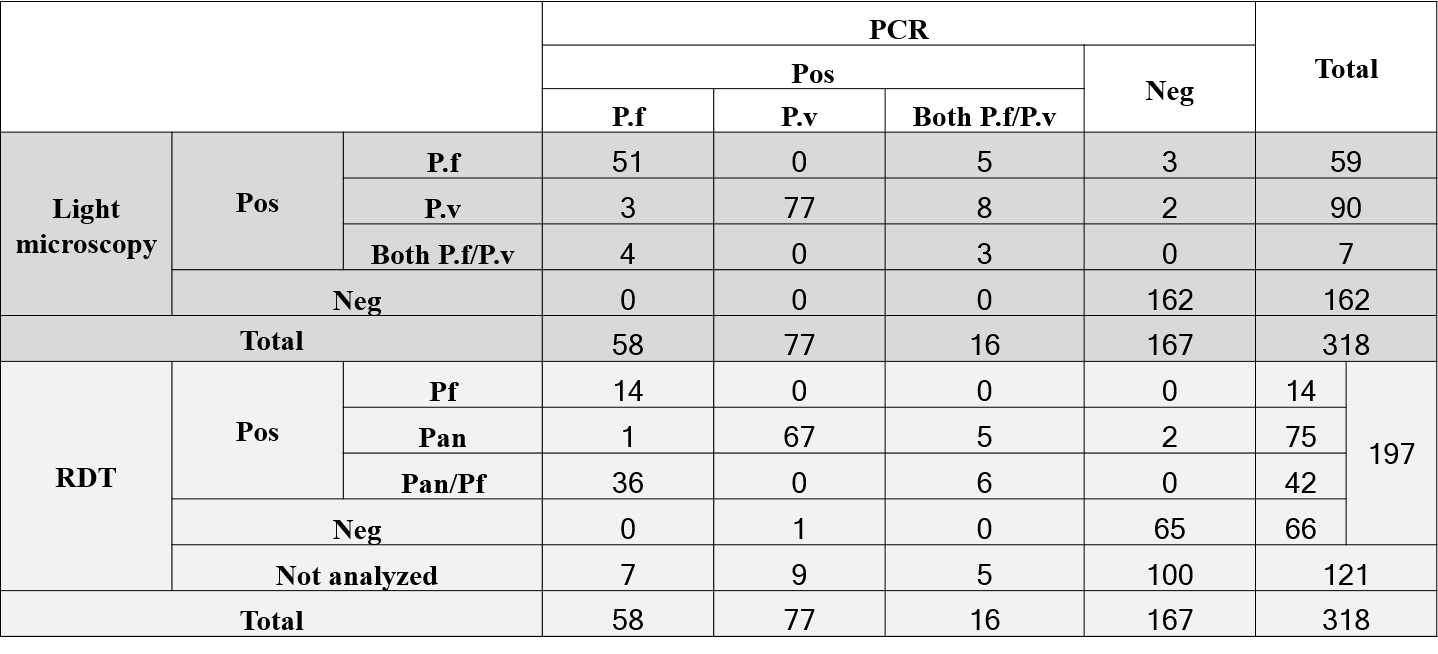


a


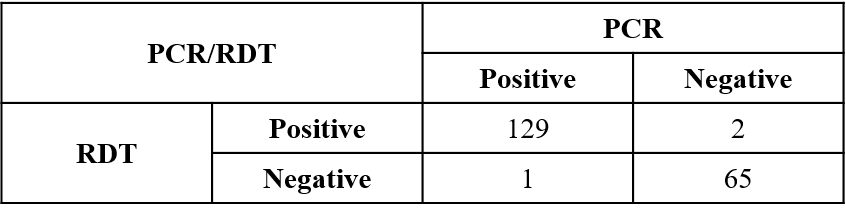


b


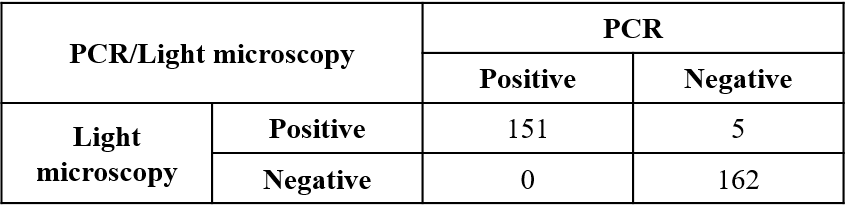


c
